# Web GIS in practice: an interactive geographical interface to English Primary Care Trust performance ratings for 2003 and 2004

**DOI:** 10.1186/1476-072X-3-16

**Published:** 2004-07-28

**Authors:** Maged N Kamel Boulos

**Affiliations:** 1 School for Health, University of Bath, Claverton Down, Bath BA2 7AY, UK

## Abstract

**Background:**

On 21 July 2004, the Healthcare Commission  released its annual star ratings of the performance of NHS Primary Care Trusts (PCTs) in England for the year ending March 2004. The Healthcare Commission started work on 1 April 2004, taking over all the functions of the former Commission for Health Improvement , which had released the corresponding PCT ratings for 2002/2003 in July 2003.

**Results:**

We produced two Web-based interactive maps of PCT star ratings, one for 2003 and the other for 2004 , with handy functions like map search (by PCT name or part of it). The maps feature a colour-blind friendly quadri-colour scheme to represent PCT star ratings. Clicking a PCT on any of the maps will display the detailed performance report of that PCT for the corresponding year.

**Conclusion:**

Using our Web-based interactive maps, users can visually appreciate at a glance the distribution of PCT performance across England. They can visually compare the performance of different PCTs in the same year and also between 2003 and 2004 (by switching between the synchronised 'PCT Ratings 2003' and 'PCT Ratings 2004' themes). The performance of many PCTs has improved in 2004, whereas some PCTs achieved lower ratings in 2004 compared to 2003. Web-based interactive geographical interfaces offer an intuitive way of indexing, accessing, mining, and understanding large healthcare information sets describing geographically differentiated phenomena. By acting as an enhanced alternative or supplement to purely textual online interfaces, interactive Web maps can further empower organisations and decision makers.

## Background

On Wednesday 21 July 2004, the Healthcare Commission  released its annual star ratings of the performance of NHS Primary Care Trusts (PCTs) in England for the year ending March 2004. The Healthcare Commission started work on 1 April 2004, taking over all the functions of the former Commission for Health Improvement , which had released the corresponding PCT ratings for 2002/2003 in July 2003 [1].

A star rating scheme is adopted. PCTs with the highest levels of performance in the measured areas are awarded a rating of three stars. PCTs with mostly high levels of performance, but which are not consistent across all measured areas, are awarded a rating of two stars. PCTs where there is some cause for concern about particular areas of measured performance are awarded a rating of one star. PCTs that have shown the poorest levels of measured performance or little progress in implementing clinical governance receive a rating of zero stars [2].

The performance ratings Web pages of the Healthcare Commission and the former Commission for Health Improvement offer a very limited "geographic search" restricted to browsing results by Strategic Health Authority (SHA)  and . This "geographic search" does not allow any visual appreciation of PCT performance levels, or any visual comparisons to be made between PCTs or between 2003 and 2004 result sets.

## Results

**Figure 1:**
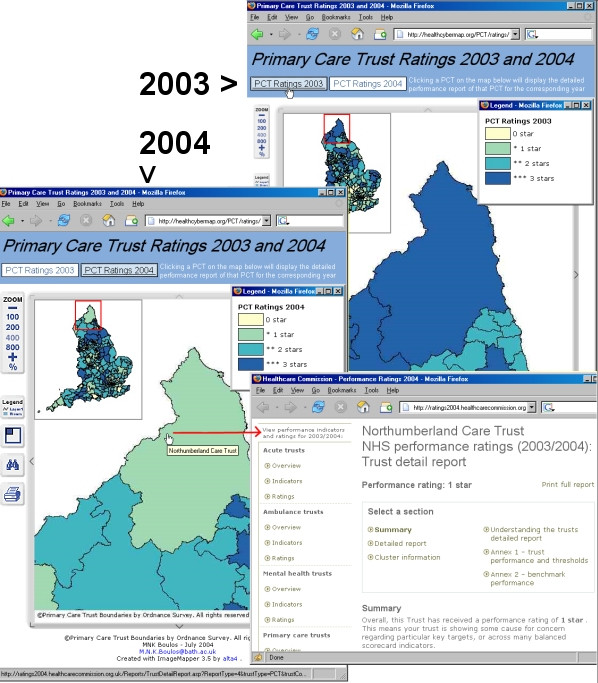
**Screenshots from our Web-based interactive maps of PCT star ratings for 2003 and 2004. **Screenshots from our Web-based interactive maps of PCT star ratings for 2003 and 2004 . When users switch between 'PCT Ratings 2003' and 'PCT Ratings 2004', the corresponding tile from the other theme is always displayed within the same pane, allowing instant 2003–2004 visual comparisons to be made. In this Figure, Northumberland Care Trust can be seen achieving lower ratings in 2004 (1 star–light green) compared to 2003 (3 stars–dark blue). The detailed 2004 performance report of Northumberland Care Trust (from the Web site of the Healthcare Commission) has been displayed by clicking the Trust shape on the 'PCT Ratings 2004' map. Note the overview maps displaying the position of the current map tile on a miniature complete map of England.

**Figure 2:**
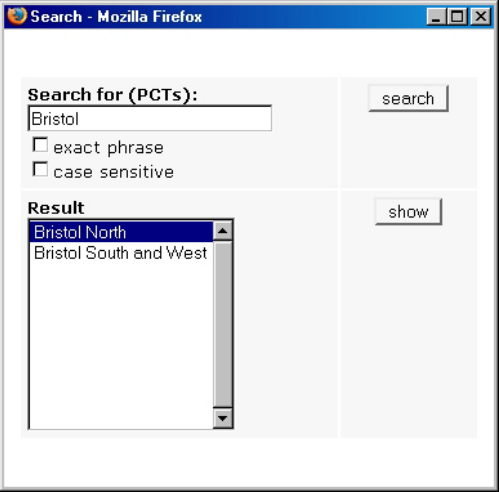
**Screenshot of the map search box. **Screenshot of the map search box, which allows users to locate a Trust on the maps by typing the Trust name or part of it. Selecting a PCT from the 'Result' list and clicking 'show' will zoom into and display the corresponding map tile for that PCT.

We produced two Web-based interactive map sets of PCT star ratings, one for 2003 and the other for 2004 . The maps use a yellow-green-blue quadri-colour scheme to represent PCT star ratings. Users can switch between the two map sets or themes, 'PCT Ratings 2003' and 'PCT Ratings 2004', within the same pane (the two themes are synchronised, so that when users switch themes the corresponding tile from the other theme is always displayed – Figure 1). Map zooming (100% to 800%), panning, MapTips (displaying PCT names), and legends are available. Dynamic overview maps are offered as navigational help (at zoom levels 200%-800%). Map search is also possible (by PCT name or part of it – Figure 2). Clicking a PCT on any of the maps will display the detailed performance report of that PCT for the corresponding year. Printer-friendly versions of the maps can be generated for direct printing from the Web browser.

The maps were successfully tested in both Microsoft Internet Explorer and Mozilla Firefox  Web browsers.

## Discussion

Many people are more visually oriented and find that spending long hours browsing the flat textual indices of the Internet leaves a lot to be desired, especially when it comes to navigating large online datasets and understanding the relationships, patterns and trends buried in them. Information resources and large textual datasets (like the detailed PCT performance reports in our case–more than 600 PCT reports for 2003 and 2004 combined) can be organised and navigated based on their geographical attributes [3]. These geographical aspects of textual information are sometimes very useful as an index to information, providing an intuitive way of accessing, mining, and understanding it. Some information types like PCT performance ratings lend themselves very well and naturally to geographical indexing and visualisation. In fact, PCT performance ratings describe a geographically differentiated phenomenon, which is the variation in the performance and quality of primary healthcare services between different areas across England.

Using our Web-based interactive maps, users can quickly and intuitively locate any PCT and retrieve detailed performance information about it. They can also visually appreciate at a glance the distribution of PCT performance across England; for example, one can instantly note that there were no three star (dark blue) PCTs in the London region in 2002/2003 and that this has remained unchanged in 2003/2004. Users can visually compare the performance of different PCTs in the same year and also between 2003 and 2004 (by switching between 'PCT Ratings 2003' and 'PCT Ratings 2004' themes). The performance of many PCTs has improved in 2004, whereas some PCTs, e.g., Northumberland Care Trust – Figure 1, achieved lower ratings in 2004 compared to 2003.

## Conclusions

Web-based interactive geographical interfaces offer an intuitive way of indexing, accessing, mining, and understanding large healthcare information sets describing geographically differentiated phenomena, and can act as an enhanced alternative or supplement to purely textual online interfaces. Geographical interfaces enable instant visual comparisons to be made between different geographical areas and over time (when information sets and maps for successive periods of time are available), thus empowering organisations and decision makers.

## Methods

Star ratings of English PCTs for the years 2002/2003 and 2003/2004 were obtained from the Web sites of the former Commission for Health Improvement  and the Healthcare Commission  respectively. The Internet addresses (URLs) of the corresponding detailed reports of PCT performance were also harvested from the same sources.

The maps were created in ESRI ArcView GIS Version 3.1 . We used the 2001 Census PCT (post April 2002 change) boundary dataset, which is the copyright of the Crown/Ordnance Survey , and is freely available to the UK academic community from EDINA UKBORDERS service with the support of the ESRC and JISC . The names/boundaries and labels (codes) of few PCTs changed between 2003 and 2004, but this was properly cared for in our exercise.

We inserted four new fields in the original PCT boundary dataset table to store the 2003 and 2004 star ratings and corresponding detailed report URLs for all English PCTs. The PCTs in the output maps are coloured according to the values in their star rating fields (0, 1, 2, or 3 corresponding to the number of stars awarded), with light colours for low ratings to dark colours for high ratings.

**Figure 3:**
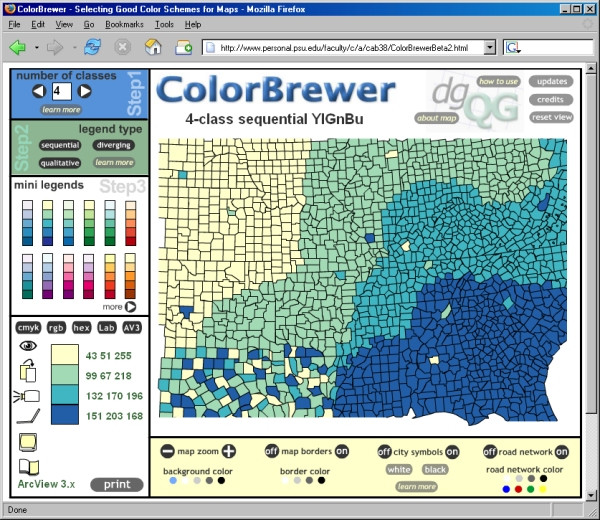
**Screenshot of ColorBrewer online tool. **Screenshot of ColorBrewer online tool  showing the colour scheme we have chosen for our maps. This yellow-green-blue quadri-colour scheme is colour blind friendly, black and white photocopy friendly (for printed output), LCD projector friendly, laptop (LCD) friendly, CRT screen friendly, and colour printing friendly–all at the same time. The corresponding Hue-Saturation-Value numerical triplets for the four colours in our scheme are also shown, ready for using in ArcView 3.x.

We used ColorBrewer  and [4]) to select a suitable colour scheme for our maps. Our chosen scheme is colour blind friendly, black and white photocopy friendly (for printed output), LCD projector friendly, laptop (LCD) friendly, CRT screen friendly, and colour printing friendly–all at the same time (Figure 3).

**Figure 4:**
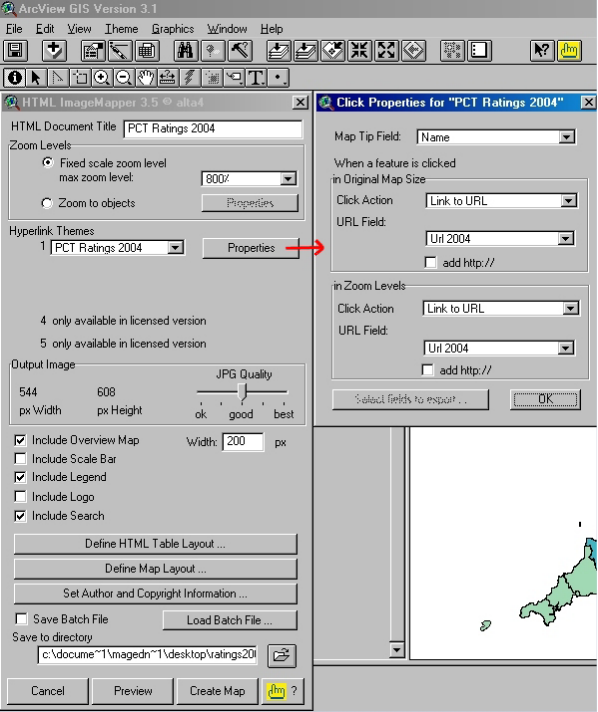
**Screenshot of the Demo version of HTML ImageMapper 3.5 extension within ArcView GIS 3.1. **Screenshot of the evaluation version of alta4 HTML ImageMapper 3.5 extension within ESRI ArcView GIS 3.1, showing the main settings we have used to generate our Web-based 'PCT Ratings 2004' interactive map set. The dialogue box on the right shows the 'MapTip Field' and 'Click Action/URL Field' settings associated with features (PCTs) on the output map.

The online interactive maps were then produced using the Demo version of alta4 HTML ImageMapper 3.5 extension for ESRI ArcView GIS 3.x  and Figure 4), and its companion tool alta4 ThemeBrowser 1.0. ThemeBrowser is used to combine separate HTML ImageMapper projects (in our case 'PCT Ratings 2003' and 'PCT Ratings 2004' map sets) into a single ThemeBrowser Web page (see ).

It is noteworthy that HTML ImageMapper does not require any server side software installation, and as such is much simpler to use than some other Internet GIS solutions like the client/server version of ALOV Map/TimeMap .

The standalone versions of ALOV Map/TimeMap  and JShape  Java applets, which don't require any server side setup, are limited by the fact that they need to download the whole map shapefile from the Web server before they can start on the client side, and so are not suitable for large datasets (our PCT boundary dataset in ESRI shapefile format is about 50 MB in size). Other options for generating interactive Web maps from a desktop GIS are discussed in [5].
